# Staphylococcus Auricularis Endocarditis: A Rare Cause of Subacute Prosthetic Valve Endocarditis with Severe Aortic Stenosis

**DOI:** 10.7759/cureus.12738

**Published:** 2021-01-16

**Authors:** Edward T Ha, John F Heitner

**Affiliations:** 1 Internal Medicine, NewYork-Presbyterian Brooklyn Methodist Hospital, Brooklyn, USA; 2 Cardiology, NewYork-Presbyterian Brooklyn Methodist Hospital, Brooklyn, USA

**Keywords:** endocarditis, myocardial infarction, aortic stenosis, staphylococcus auricularis

## Abstract

Prosthetic valve endocarditis (PVE) represents 20% of all cases of endocarditis. Herein, we present a rare cause of PVE by Staphylococcus auricularis (S. auricularis) exhibiting features of subacute endocarditis causing severe aortic stenosis and acute myocardial infarction.

## Introduction

Prosthetic valve endocarditis (PVE) refers to the infection of an implanted (either surgical or transcatheter approach) prosthetic valve. PVE represents about 20% of all cases of endocarditis. The presence of a bioprosthetic valve is considered a high-risk factor for endocarditis occurring in 1%-6% of patients with prosthetic valves [1], with an annual incidence estimated to be 0.3% to 1.2% per patient-year [2]. It usually affects the left side of the heart and has an equal predilection for both aortic and mitral valves [3,4]. The typical presentation of bacterial endocarditis is that of an infectious etiology, with fevers present in 70% of the cases [5], and additional non-specific symptoms may include chills, anorexia, weight loss, malaise, headache, myalgias, arthralgias, night sweats, abdominal pain, dyspnea, cough, and pleuritic pain.

The microbiology and pathology of PVE is rather predictable, which depends on the time of presentation from the date of implantation. Early infection, defined as those occurring up to one year of implantation, typically involves Staphylococcus aureus, Staphylococcus epidermidis, and streptococci species. Early infections typically seed during implantation and can affect the structural components, such as the valve sewing ring, cardiac annulus, and anchoring sutures causing paravalvular abscesses. In contrast, late infections are more likely to involve the Streptococcus species in addition to Staphylococcus aureus and typically affect the valvular leaflets. The presence of heart failure complicating PVE is also a common phenomenon, ranging in 30%-60% of patients [2,3]. To aid in the diagnosis, echocardiography should be obtained in all patients suspected of PVE. The most common abnormalities on echocardiogram would be vegetations and/or abscess on the affected valve present in 70% and 15%-30% of patients, respectively [2,5]. New valvular regurgitant lesions were seen in as much as 70% of patients [2]. Bacterial endocarditis caused by Staphylococcus auricularis (S. auricularis) presenting with severe aortic stenosis and type-2 non-ST-segment elevation myocardial infarction (NSTEMI) is an exceptionally rare entity with no known documented cases.

## Case presentation

A 77-year-old male with a past medical history of hypertension, non-ischemic cardiomyopathy, ejection fraction (EF) of 25%, status-post implantable cardioverter defibrillator, severe aortic regurgitation with myxoid degeneration of aortic valve status-post surgical repair three years prior to admission, and cerebral vascular accident (with residual left-sided weakness) presented to our emergency department with worsening shortness of breath at rest, associated with a productive cough for the past three days. 

His physical exam was significant for moderate respiratory distress (respiratory rate 28), bilateral inspiratory crackles in the lung bases, elevated jugular venous distention (8 cm) and positive hepatojugular reflex, and significant left-sided upper and lower extremity weaknesses. 

**Figure 1:**
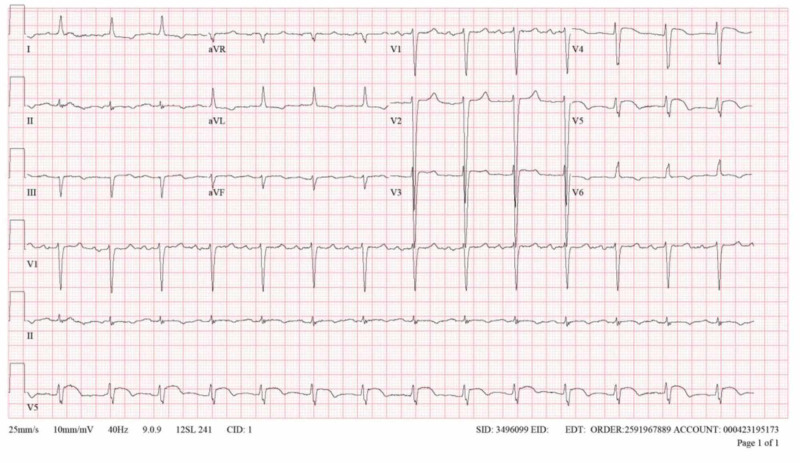
Admission electrocardiogram demonstrating ST-segment elevation in precordial leads

The patient’s labs were remarkable for a white blood cell count of 19,900 cells/uL (reference range, 4,000 to 10,300 cells/uL), absolute neutrophil count of 18,200 cells/uL (reference range, 1,400 to 7,000 cells/uL), hemoglobin of 9.1 g/dL (reference range, 12,500 to 16,900 g/dL), erythrocyte sedimentation rate (ESR) of 100 MM/hr (reference range, 0 to 20 MM/hr), C-reactive protein (CRP) of 110 mg/L (reference limit of normal, < 3 mg/L), procalcitonin of 0.8 ng/mL, blood urea nitrogen of 56 mg/dL (reference range, 7 to 18 mg/dL), creatinine of 2.32 mg/dL (reference range, 0.67 to 1.67 mg/dL), potassium of 5.4 mmol/L (reference range, 3.5 to 5.1 mmol/L), troponin I of 60.8 ng/mL (reference limit of normal < 0.045 ng/mL), pro-B-type natriuretic peptide of 174,109 pg/mL (reference limit of normal < 450 pg/mL), and lactate of 1.35 mmol/L (reference range, 0.36 to 1.25 mmol/L). His chest X-ray revealed cardiomegaly and mild pulmonary edema, a minimal right pleural effusion, and a possible superimposed patchy infiltrate in the right mid-lung field. Electrocardiogram indicated normal sinus rhythm, poor R-wave progression, voltage criteria for left ventricular hypertrophy (Cornell Criteria), and ST-segment elevation in the three contiguous precordial leads V3-V5 without reciprocal changes (Figure 1). At this point, differential diagnoses included acute heart failure exacerbation secondary to pneumonia and/or acute myocardial infarction. 

In the emergency department, blood cultures were obtained, and the patient was treated with ceftriaxone/azithromycin, aspirin, ticagrelor, and heparin. An emergent left-heart catheterization was not performed due to transient ST-segment changes more consistent with conduction abnormalities and lack of typical chest pain. A trans-thoracic echocardiogram was performed which revealed left ventricular ejection fraction (LVEF) 20% with severe diffuse hypokinesis of the ventricular walls, stage three diastolic dysfunction, and a possible 8 mm apical wall thrombus. In addition, the bioprosthetic aortic valve was severely stenotic (mean gradient 19.5 mmHg in the setting of heart failure and valve area 0.74 cm^2^), as a result of a mobile vegetation measuring 19 mm, which was better visualized on a subsequent transesophageal echocardiogram (Videos 1-3). Shortly thereafter, five out of six blood cultures grew S. auricularis. The patient was treated with the appropriate antibiotics based on sensitivities and subsequently discharged home with IV antibiotics.

**Video 1:** Transesophageal echocardiographic (mid-esophageal, aortic valve, short-axis view) video clip demonstrating 2 cm aortic valve vegetation

**Video 2:** Transesophageal echocardiographic (mid-esophageal, aortic valve, long-axis view) video clip demonstrating 2 cm aortic valve vegetation

**Video 3:** Transthoracic echocardiographic (apical 5 chamber view) Doppler video clip across the aortic valve

## Discussion

This patient presented with subacute endocarditis of his bioprosthetic aortic valve secondary to inoculation with S. auricularis. S. auricularis was discovered in 1983 and was found to be a colonizer of the external auditory meatus. S. auricularis is a rare form of community or nosocomial infections and even rarer cause of endocarditis with only five documented cases [6-10]. Our case is the first to demonstrate an atypical, late-presenting, and subacute PVE resulting in severe aortic stenosis caused by S. auricularis vegetation. The patient presented with ST-segment changes on electrocardiogram and troponin I elevations as high as 60 ng/mL (reference limit of normal < 0.045 $\frac{ng}{\\mL}$), concerning for acute coronary syndrome. The mechanism for the myocardial infarction is unclear, but may be multifactorial, in that it can be explained by a lack of adequate coronary perfusion pressures from systolic dysfunction and increased left ventricular pressures in severe aortic stenosis causing impaired relaxation, and increased oxygen demand from catabolic state of his indolent infection. Another possibility is embolization of a portion of the vegetation, though no regional wall motion abnormalities were found on echocardiogram. To our knowledge, only two other cases of severe aortic stenosis presenting as acute coronary syndrome have been reported [11,12].

Our patient was found to have severe aortic stenosis of his prosthetic valve secondary to a 2 cm mobile, infectious vegetation. Although there is a possibility that the stenotic valve was a nidus for infection, this is unlikely given that the valve was three years old. Valvular stenosis secondary to bacterial endocarditis is also an uncommon entity, documented mostly in patients with a prior history of rheumatic disease. Those involving a prosthetic valve has been due to fastidious or less virulent organisms, particularly Bartonella species [13-18]. This is the first case to document S. auricularis causing late PVE, where it exhibits a pattern of growth presenting without overt symptoms of sepsis, similar to fastidious organisms. Therefore, it allows the forming of a clinically significant vegetation that cause valvular stenosis. Given the low virulence and rarity of this particular organism, it is unclear when the patient was inoculated with this organism given that his baseline echocardiogram performed one and a half years post-implantation was a technically difficult study unable to visualize the aortic valve. 

PVE is associated with a one-year mortality rate as high as 30% in one cohort [19]. According to the International Collaboration on Endocarditis-Prospective Cohort Study risk score which predicts outcomes in those with infective endocarditis, our patient’s score correlates with a 20% risk of death at the six-month mark [20]. Given the high mortality associated with PVE, early detection of subacute PVE before overt signs of cardiovascular decompensation may lead to improved outcomes in this high-risk cohort.

## Conclusions

Sub-acute PVE endocarditis by S. auricularis can present without overt signs of sepsis. The clinician and cardiologist should consider PVE due to fastidious organisms in the differential of a patient with subtle signs of chronic inflammation such as leukocytosis, elevated ESR/CRP, in the absence of fever and myalgia. Serial echocardiograms done in the outpatient setting have the potential to detect sub-acute PVE early before the vegetation causes significant cardiovascular morbidity and mortality. We recommend early and frequent echocardiograms after implantation of a prosthetic valve to aid in the detection of PVE, especially in high-risk patients such as those with baseline heart failure. 

## References

[1] Østergaard L, Valeur N, Wang A (2019). Incidence of infective endocarditis in patients considered at moderate risk. Eur Heart J.

[2] Wang A, Athan E, Pappas PA (2007). Contemporary clinical profile and outcome of prosthetic valve endocarditis. JAMA.

[3] Ivert TSA, Dismukes WE, Cobbs CG, Blackstone EH, Kirklin JW, Bergdahl LA (1984). Prosthetic valve endocarditis. Circulation.

[4] Hammermeister KE, Sethi GK, Henderson WG, Oprian C, Kim T, Rahimtoola S (1993). A comparison of outcomes in men 11 years after heart-valve replacement with a mechanical valve or bioprosthesis. N Engl J Med.

[5] Amat-Santos IJ, Messika-Zeitoun D, Eltchaninoff H (2015). Infective endocarditis after transcatheter aortic valve implantation: results from a large multicenter registry. Circulation.

[6] Amir R, Mandalaparty C, DeHart D (2020). Contaminant or culprit”: a novel case of Staphylococcus auricularis endocarditis. J Am Coll Cardiol.

[7] Williford S, Heavner M, Lambing T, Wian B, Ma S, Gonzales J (2018). When “contaminants” become pathogens: Staphylococcus auricularis bacteremia in the critically ill. Crit Care Med.

[8] Hoffman DJ, Brown GD, Lombardo FA (2007). Early-onset sepsis with Staphylococcus auricularis in an extremely low-birth weight infant - an uncommon pathogen. J Perinatol.

[9] Lew SQ, Saez J, Whyte R, Stephenson Y (2004). Peritoneal dialysis-associated peritonitis caused by Staphylococcus auricularis. Perit Dial Int.

[10] Stojanović P, Kocić B, Randelović G, Cirić V (2008). Coagulase-negative Staphylococcus isolated from bloodculture: causes or contaminants?. Med Pregl.

[11] Wayangankar SA, Dasari TW, Lozano PM, Beckman KJ (2010). A case of critical aortic stenosis masquerading as acute coronary syndrome. Cardiol Res Pract.

[12] Gue YX, Bhandari SS, Kelly DJ (2017). Critical aortic stenosis presenting as STEMI. Echo Res Pract.

[13] Copeland JG, Salomon NW, Stinson EB, Popp RL, Shumway NE (1979). Acute mitral valvular obstruction from infective endocarditis: echocardiographic diagnosis and report of the second successfully treated case. J Thorac Cardiovasc Surg.

[14] Sacks PV, Lakier JB, Barlow JB (1969). Severe aortic stenosis produced by bacterial endocarditis. Br Med J.

[15] Roberts WC, Ewy GA, Glancy DL, Marcus FI (1967). Valvular stenosis produced by active infective endocarditis. Circulation.

[16] Davies MK, Ireland MA, Clarke DB (1981). Infective endocarditis from group C streptococci causing stenosis of both the aortic and mitral valves. Thorax.

[17] Kreisel D, Pasque MK, Damiano RJ Jr, Medoff G, Kates A, Kreisel FH, Lawton JS (2005). Bartonella species-induced prosthetic valve endocarditis associated with rapid progression of valvular stenosis. J Thorac Cardiovasc Surg.

[18] Schnitzer K, Or Z, Sawaed S, Sharoni E, Bisharat N (2017). Rapidly progressive bioprosthetic aortic valve stenosis due to Bartonella species endocarditis. Ann Thorac Surg.

[19] Nishimura RA, Otto CM, Bonow RO (2014). 2014 AHA/ACC guideline for the management of patients with valvular heart disease: executive summary. Circulation.

[20] Park LP, Chu VH, Peterson G (2016). Validated risk score for predicting 6-month mortality in infective endocarditis. J Am Heart Assoc.

